# Regenerative medicine meets mathematical modelling: developing symbiotic relationships

**DOI:** 10.1038/s41536-021-00134-2

**Published:** 2021-04-12

**Authors:** S. L. Waters, L. J. Schumacher, A. J. El Haj

**Affiliations:** 1 Oxford Centre for Industrial and Applied Mathematics, Mathematical Institute, Radcliffe Observatory Quarter, University of Oxford, Oxford, UK; 2 Centre for Regenerative Medicine, The University of Edinburgh, Edinburgh BioQuarter, Edinburgh, UK; 3 Healthcare Technology Institute, Institute of Translational Medicine, School of Chemical Engineering, University of Birmingham, Birmingham, UK

**Keywords:** Translational research, Stem-cell therapies

## Abstract

Successful progression from bench to bedside for regenerative medicine products is challenging and requires a multidisciplinary approach. What has not yet been fully recognised is the potential for quantitative data analysis and mathematical modelling approaches to support this process. In this review, we highlight the wealth of opportunities for embedding mathematical and computational approaches within all stages of the regenerative medicine pipeline. We explore how exploiting quantitative mathematical and computational approaches, alongside state-of-the-art regenerative medicine research, can lead to therapies that potentially can be more rapidly translated into the clinic.

## Introduction and vision

The concept of using regenerative medicine approaches to repair and regenerate tissue damaged through disease or trauma has been maturing over the past few decades. Translation of regenerative therapies to the patient – bench-to-bedside – is one of the global multidisciplinary challenges of our time, offering a vision of new therapies with the power to address major unmet healthcare needs^1^.

Regenerative therapies can utilise progenitor or stem cells which are delivered to a repair site or area of degeneration to restore tissue structure and function. Regenerative therapies may also include a molecule or biomaterial based approach which promotes endogenous recruitment and tissue repair. Key examples of the challenges which regenerative therapies are facing include: choice of the best cell type from multiple sources, both autologous and allogeneic, or adult and embryonic^2^; new ways for manufacturing therapeutic doses of donor stem cells which are characterised as Advanced Therapy Manufacturing Platforms (ATMPs); new enabling technologies using optical, sensing and mechanical tools for routine use to support scaled up cell production^3^; novel biomaterials providing structural tissue mimics and instructive cues based on topography and protein chemistry; 3D tissue models grown in bioreactors or growth chambers presenting new ways for testing potential therapeutic strategies before implantation; optimising clinical choice and patient stratification using cell-based assays which aim to improve efficacy, and long term outcomes in patients.; and finally, patient monitoring at cell resolution using MRI, PET and multi-modal approaches which support efforts to move to clinical first in man and 1st stage trials^4^.

These broad challenges which aim to advance a new type of medicine have relied on multiple disciplines covering cell biology to material chemistry, enabling physics and clinical medicine. It is now being recognised that additional maths-based approaches may speed the advance of these therapies and enable them to reach the clinic faster. One example of such an approach is in silico modelling. This disruptive perspective can bring mathematicians into the pathway at many stages of the translation; mechanistic modelling studies enable acceleration of translational research by optimisation of protocols, new algorithms and statistics help to define our quantitative metrics and new data science and AI innovations expand our use of patient derived databases to optimise therapies.

A key challenge in integrating mathematical approaches into the regenerative medicine pathway is to identify where mathematical modelling can make the most disruptive impact. Mathematical modelling approaches can be much faster and cheaper than performing numerous time consuming and expensive laboratory experiments^5^. Embedding mathematics within regenerative medicine enables researchers to go beyond the usual trial-and-error approach, be guided in their experimental design, and therefore accelerate advances in regenerative medicine^6^. Mathematical models provide mechanistic insight into complex biological systems exhibiting richly non-linear behaviour, and predictions from mathematical models can be used to optimise protocols both for the manufacture of regenerative medicine products as well as for treatment strategies, e.g. the delivery of cell therapies. Mathematical models that predict the dynamic behaviour of the regenerative product, e.g. tissue growth during in vitro culture, can potentially be used as online monitoring tools to ensure the reproducibility and safety of manufactured products, addressing challenges in product regulation. Finally, bespoke patient models may be built in an individualised medicine approach and these models can be used to predict the efficacy of regenerative medicine strategies^7^.

Mathematical models have traditionally been used to provide mechanistic insight into the many interactions between the biological components of a system, for example enabling quantitative assessment of the cellular microenvironment that can then be manipulated to guide cell behaviour during development or growth. By systematically varying the parameters of the models, or by the addition of new components, we can perturb the model leading to new predictions and insights that can be used to overcome bottlenecks. For example, understanding gained from in vitro systems can be translated to the in vivo scenario through the inclusion of an immune component in the in silico models^8^. Models can also be used to “bridge the gap” between sub-disciplines by integrating multiple quantitative data sources such as imaging and molecular or biomechanical data, for example^9^.

### A brief introduction to common modelling approaches

**Fig. 1:**
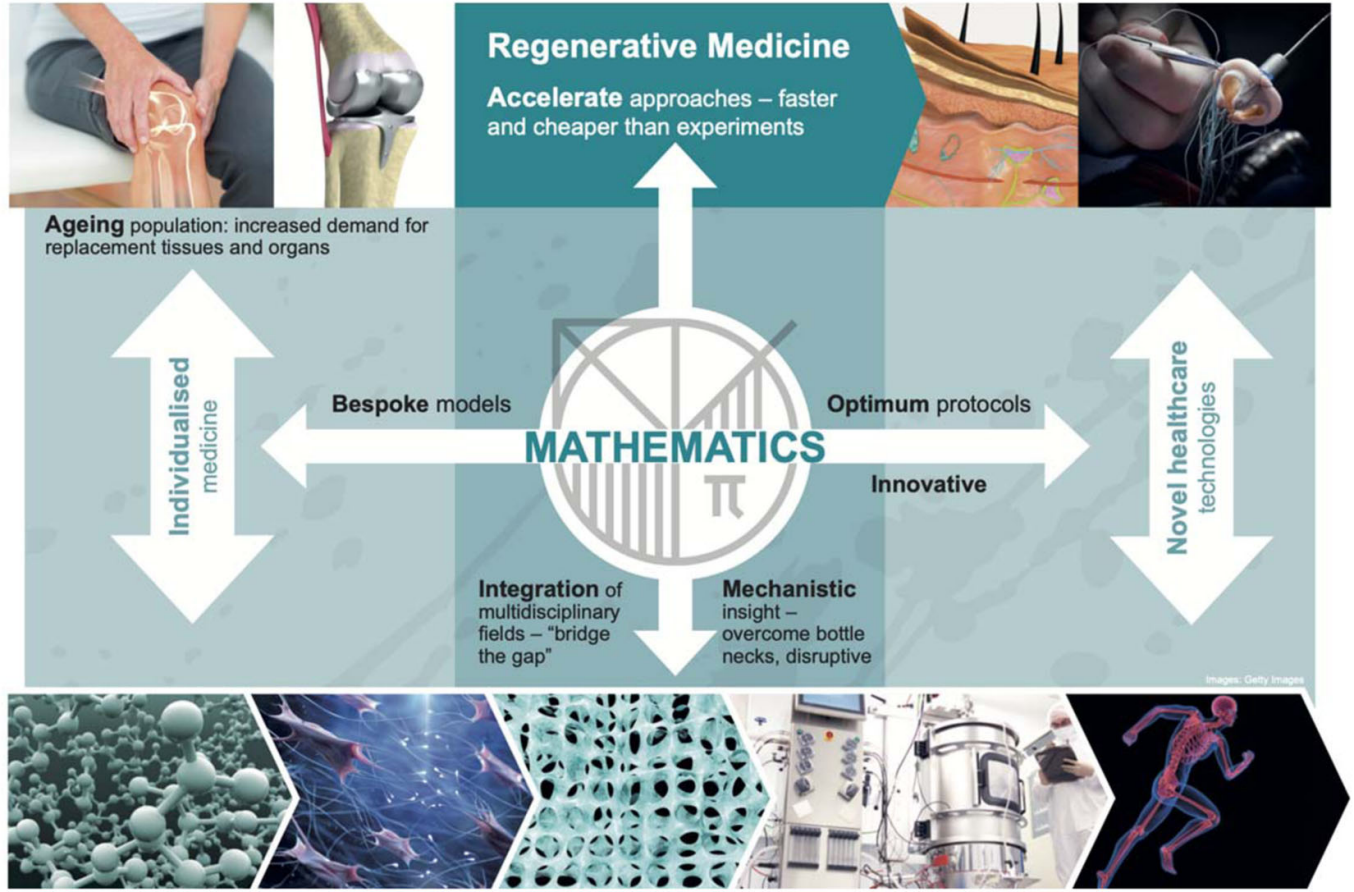
A new era of symbiosis between regenerative medicine and maths. Diagram which highlights the many opportunities for utilising mathematical and computational approaches within regenerative medicine. This figure was created for the authors by the University of Edinburgh’s graphic design service team.

Multicellular, multiscale biological systems can often be too complex to understand by interrogating experimental and clinical data with verbal thinking and linear reasoning alone, thus the addition of theoretical or in silico models, expressed in the precise and powerful language of mathematics, can provide new and deeper insights. The key steps in the development of mathematical models are model construction, calibration, prediction, and refinement. We discuss the choices to be made in model construction at greater length below. Briefly, theoretical models may be phenomenological or mechanistic and describe biological processes at different scales: on the whole patient, organ, tissue scale, single-cell-level, and even the molecular level (Fig. 1).

**Fig. 2:**
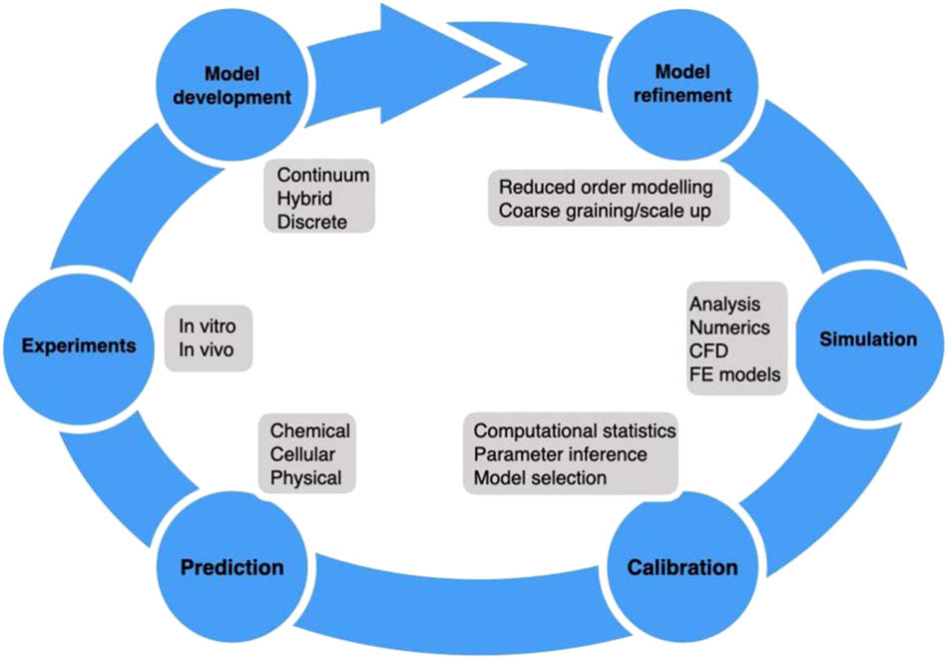
Methodology. An illustrative diagram showing the quantitative regenerative medicine pipeline with stages of the modelling process and types of modelling involved.

A key aspect in the development of biologically realistic, predictive mathematical models, is interfacing mathematical models with experimental data. The calibration of models through comparison of model outputs with experimental data poses additional formidable challenges because the available data are usually complex, high-dimensional, noisy, and often incompletely observed. Comparing models with data is vital for parameter inference, which is the inverse problem of determining which parameter values are most likely to produce the observed data. Another reason to compare models with data is for the purpose of model selection, i.e. determining the level of model complexity required to interrogate a given set of experimental data, or deciding which model out of several competing hypotheses is more likely to be true. Once calibrated, the theoretical models are validated via detailed comparison of mathematical model predictions with experimental data. The use of predictions, whether on existing and withheld data, or predictions that are to be tested by newly generated data, are a key aspect of any mathematical modelling process. Any discrepancies between model predictions and experimental data can then lead to further model refinement. An iterative cycle of predict-test-refine is fundamental to the development of all models (See Fig. 2).

**Table 1:** Key challenges for RM where new approaches can make a major shift in translation.

	Engineering cells and tissues	Validation in vitro and in vivo	Manufacture and Validation	Define Measure and Control	Clinical cohorts and optimisation
Enabling science, technology	Mechano- biology; cell differentiation,co-cultures; cell substrate niche interactions; biomaterial development	3D models, bioreactors, animal models of disease, ex vivo models,	Bioreactors, processing, scale up, scale out, cell separation and delivery tools	Non-invasive imaging, sensor design, Biomarker Analysis	Trial design, Defining patient groups, Feedback maths analysis, Data analytics
Product development	Stem cell control systems/ biomaterial delivery	Advanced Therapy Medicinal Products, Cell free implants, regenerative factors,	Autologous cell therapy scale up, Regenerative products	Novel sensors, Metrology	Trial design tools, Patient data handling
Clinical relevance and translation	Iterative first in man, Trials in identified cohorts	Optimised therapies, Human Cell sources, Regulatory confidence	Minimally invasive, delivery devices, validation models	Tracking of implanted cells, new outcome measures	Criteria for adoption, health economics, patient and public involvement (PPI)

The choice of modelling approach is guided by the biological question being asked, and the nature of the quantitative experimental data (see Table 2). Here we give a brief and broad categorisation of commonly used types of mathematical modelling in biology and medicine.

- *Mechanistic models:* Mechanistic models represent all the components of a hypothesis (cell-cell interactions, role of biomolecules on cell behaviour, etc.) mathematically^10^. Mechanistic model development is often guided by analysis of experimental data, allowing hypotheses to be made for the causal mechanisms underlying a biological system^5^. For example, in describing the growth of a mechanosensitive tissue such as bone in a bioreactor, causal mechanisms include the response of the mechanosensitive cells to the applied mechanical load (fluid shear stress, hydrostatic pressure, substrate deformation etc). A key step is to identify the dependent variables of the system e.g. cell number, fluid velocity, substrate density, and their dependence on the independent variables of the system e.g. space and time.

**Table 2:** Next steps for future links to mathematical teams.

	I want to model…	My data are…	Consider this kind of maths	Example refs
Basic regenerative biology	Gene regulation & transcriptional control	Transcriptomics, time-course of gene/protein expression, live imaging of gene/protein expression	Differential equations for e.g. concentration of mRNA, stochastic models for control of gene expression by proteins	^69–71^
	Cell migration	Cell tracking, population snapshot, time-course of population	Cell-based, differential equations, statistical	^17,18,26,27^
	Cell-cell signalling	Cell counts, time-course of cell number, signalling molecule concentration	Differential equations, hybrid models	^37–40,62,64^
Relevant to bioreactors	Lineage choice, differentiation, cell reprogramming	Clonal tracking, live imaging of cell phenotype, transcriptomics, epigenomics, metabolic activity	Stochastic and deterministic differential equation models of cell division & differentiation	Relevant models from different applications:^19,28–30,72^
	Pattern formation, growth	High-content microscopy images/movies	Cell-based, differential equations	^9,11,14,31,32,41,46–48,50,51,53–58,73^
	Bioreactor optimisation	Flow rates, inlet and outlet solute concentrations, cell distributions	Differential equations	^10,51,59,61,74^
Clinical/ translational	Manufacturing	Online monitoring label-free impedance (electrical)	Differential equations	^60,74^
	Trial optimisation	In silico pre-clinical patient-based clinical cohorts	Statistical machine-learning optimal control theory	^21,22,61,63,67^
	Efficacy and safety	Toxicity, immunogenicity	Differential equations, statistical	^64–66,75^

Mechanistic models can be multi-scale, incorporating processes on a range of spatial and temporal scales. The development of coarse-graining methods for models that contain disparate space and time scales is crucial to enable rigorous mathematical analysis, and for general classification of models according to their predicted emergent behaviours^11,12^. Efficient and accurate computational methods for simulation of multi-scale mathematical models^13^ are necessary to enable full investigation of potential model behaviours, parameter sensitivity analysis, and data-driven model calibration.

The representation of a biological mechanism need not be reductionist and molecular, but can be phenomenological^14^. Phenomenological models aim to reproduce the experimental observations without the terms in the model equations necessarily corresponding to cellular or molecular processes directly. An example is a model of a homoeostatic epithelium in which cell division always co-occurs with another cell dying or migrating away^15^, so that the overall cell density stays constant (which is the important phenomenology to capture). Phenomenological models, despite what the name suggests, can still provide mechanistic understanding of a system’s function, for example what kind of regulatory interactions are important, even if the precise nature of the interactions and their molecular mediators remains obscure. In such cases, there may be many underlying molecular mechanisms that give rise to the same phenomenological models. Strictly speaking, all models are phenomenological at some level, as they simplify the underlying chemistry and physics considerably. The model development process for phenomenological models is largely the same as for other mechanistic models described above.

Mechanistic modelling approaches include continuum^16^/discrete^17^, hybrid^18^, and deterministic/stochastic^19,20^.

- *Discrete models* treat cells as distinct entities and consider the behaviour of one or more individual cells, accounting for their interactions both with each other and with the surrounding microenvironment. Discrete cell models provide a natural framework for incorporating available quantitative experimental data at the cellular or subcellular scale. Often, discrete models are also *stochastic models*, meaning that the outcome is to some degree random, and only one of many possible realisations. Average model behaviours or a full distribution of predictions can be obtained by repeating stochastic model simulations many times^17^.
- *Continuum models* average the cell behaviour over a number of cells, for example describing the behaviour of the cell population in terms of its density that depends continuously on space and time. Continuum models are also used to describe the surrounding mechanical and chemical environment, with variables such as fluid velocity and pressure, solid deformation and solute concentration again depending continuously on space and time^10^. Hybrid discrete-continuum models refer to the integration of discrete cell-based models with continuum models for the surrounding cellular microenvironment, or the use of discrete (low cell numbers) and continuum (high cell numbers) models in different regions of the spatial domain as appropriate^18^. In a deterministic mathematical model, the spatiotemporal evolution of the dependent variables is completely determined by the model parameters and initial conditions - such a model will therefore always produce the same output for a given initial state. A natural formulation of continuum models is in the form of *differential equations*.
- *Statistical models* are another class of models which focus on prediction over mechanistic insight. Examples of statistical models are general linear models, logistic regression, and machine-learning techniques such as artificial neural networks^21,22^. Statistical models aim to fit or learn the relationship of input variables, such as experimental parameters or biological variables, to output variables, such as experimental measurements. Through this, statistical models can be used to predict how the distribution of e.g. experimental measurements should change under a change in the input variables. In the development of statistical models, data are typically divided into training, validation, and test data. Training data are used to train the model (i.e. fit its parameters), validation data are used to prevent overfitting, and test data are used to assess the model’s performance at prediction^23^. Unlike phenomenological models, in which individual model components implicitly represent biological processes, no such interpretability is offered a priori by statistical models. Interpretability is however possible by inspecting the model after training it on data, although the degree of interpretability varies depending on the statistical method used^24^.

### Mathematical models in regenerative medicine research

Mathematical approaches have traditionally focused on the discovery science end of the spectrum of regenerative medicine research. This has stemmed from a strong research base in mathematical medicine and biology where there are existing successful interactions with biologists and medics. Major questions in developmental and stem cell biology have been investigated using experimental and theoretical approaches^25–28^. Another area that has received a lot of attention is modelling of tissue growth within bioreactors, as this draws on a long tradition of continuum mechanics and its applications to medicine and biology.

### Basic regeneration biology

Models can be used to distinguish which cellular processes are important to the overall regenerative process. For example, models incorporating both cell proliferation and migration can be used to explore the contribution of each process to experimentally observed regeneration. The balance of quiescence vs proliferation has been investigated in several studies. For example, the balance of quiescence and proliferation in neural stem cells has been modelled by a compartment-based differential equation approach (a continuum model) to investigate the change in regenerative capacity due to increased quiescence with age^29^. By modelling a simplified signalling network and using single-cell RNAseq data^29^ the authors were able to identify a potential niche signal that maintains quiescence, Wnt Antagonist sFRP5. Another study investigating the balance of quiescent and proliferative cells in regeneration in liver biliary epithelial cells found little interconversion (on shorter time-scales) based on dual labelling experiments, and used a discrete, stochastic model of symmetric and asymmetric cell divisions to explain distribution of clone sizes^30^. At the larger scale of cell population dynamics, axolotl spinal cord regeneration has been modelled with compartment-based differential equations to identify that acceleration of the cell cycle is a more important part of the regenerative response than cell influx and stem cell activation^31^.

Mathematical modelling and data analysis approaches can be used to identify similarities between developmental and regenerative processes, i.e. can “developmental processes be reinstated and adapted or are there entirely new regenerative processes to be discovered?”^32^. A recent single-cell scale analysis^33^ investigated to what extent cells in axolotl limb regeneration are de-differentiating into multipotent states, and how similar these states are to their developmentally observed analogues. Another recent example using this approach of comparing cell states in single-cell sequencing data identified a “regeneration-organising cell” in Xenopus tails^34^. Another question underpinning regeneration and growth of tissues is why does regeneration occur in some animals but not others^35^? One approach may be to compare regeneration and wound healing, and what factors affect successful healing vs scarring. Similarities in gene expression between regeneration and wound healing have been identified^36^, however the complexity of the involvement of the immune system has not been mapped. Modelling could provide a means to address these additional components before carrying out a large number of complex co-culture approaches, thus guiding experiments towards the inclusion of essential components. One study^37^ used coupled differential equations to model cytokine signalling in microglia, and explained the pro- and anti-inflammatory effects of cytokine perturbations through differential kinetics in parallel negative feedback loops. This has implications for treatment e.g. of neuroinflammation/neurodegenerative associated conditions through application of cytokines.

Another example which demonstrates the utility of mathematics in defining the role of cell interactions for successful regeneration is in hair regeneration. Spatial simulations using both continuum and discrete models have shown how a collective cell behaviour akin to bacterial quorum sensing causes hair follicle regeneration in mice to occur only when the injury is large enough^38^. Other studies from the same group of authors^39,40^ use spatial discrete and stochastic modelling to show how the coupling, i.e. the strength of communication, between hair follicles determines the pattern in which hairs regenerate, e.g. in spreading waves, and why regeneration may stop in human scalps where stem cell activities may be more independent and less coupled. Further work^41^ has investigated the morphogenesis of skin layers and hair follicles in vitro from dissociated mouse epidermal and dermal cells, and thus identified crucial physical and molecular events in the process. This led to a partial rescue of hair forming ability in these reconstituted skin samples when formed from adult cells, through the timed application of growth factors, Wnts, and MMPs^41^.

### Bioreactors

Mathematical modelling of tissue maintenance and growth within in vitro bioreactors is motivated by the desire to understand and control how the imposed experimental environment and operating conditions influences the time-dependent and spatial distribution of cells, nutrients, fluid flow and substrate deformation within the bioreactor. In vitro engineering of 3D tissues is characterised by a source of cells (autologous, allogeneic, xenogenic) which are seeded on a substrate or biomaterial scaffold which can be used to provide chemical, topographical and mechanical cues^42^. Scaffolds can be extremely varied materials – synthetic e.g. or natural (decellularised ECM) – and cell-seeded scaffolds are cultured within bioreactors. Significant tissue-engineering studies have progressed the field in bone tissue engineering^43^. Examples of bioreactors include perfusion, compression, hollow fibre, hydrostatic etc^44,45^.

Mathematical models of bioreactors range from details of the fluid-tissue interaction at the pore scale within a cell-seeded scaffold^46^ to models of growing tissue constructs^47,48^. We do not present a comprehensive review of bioreactor modelling studies here, but instead highlight how mathematical modelling techniques have been applied to these systems. Recent work has shown that in addition to material scaffold properties such as surface roughness, elasticity and substrate chemistry, the macroscopic geometry of the substrate controls cell growth kinetics^49^. By using rapid prototyping to build artificial macro-pores of different controlled geometries, Rumpler et al. demonstrated that cells locally respond to high curvature through enhanced tissue growth^50^. Additionally, mechanosensitive cells respond to fluid shear stress, which is itself a function of the pore geometry. Sanaei et al.^46^ developed a continuum mathematical model for the fluid flow through an individual scaffold pore, coupled to the growth of cells on the pore walls, to determine how the interplay between substrate geometry and fluid shear stress enhances tissue growth. In a complementary approach, Guyot et al.^51^ developed a 3D computational model using the level set method to capture the growth, again depending on curvature and fluid shear stress, at the scaffold level in a perfusion bioreactor. These models offer simple frameworks for testing the behaviour of different scaffold pore geometries, and facilitates the prediction of operating regimes (inlet fluid flux etc) in which the tissue growth may be enhanced.

While computational approaches can be employed to scale-up mechanistic insights from the pore to the tissue scale, an alternative approach is to use mathematical homogenisation techniques to derive effective macroscale equations (construct level) that explicitly incorporate details of the structure and dynamics of the pore scale detail. Such *coarse-graining* approaches rely on a disparity in length scales e.g. between the pore scale and scaffold scale. A recent experimental approach to engineer artificial cartilage involves seeding cells within a scaffold consisting of an interconnected 3D-printed lattice of polymer fibres combined with a cast or printed hydrogel, and subjecting the construct (cell-seeded scaffold) to an applied load in a bioreactor^52^. To understand how the applied load is distributed throughout the construct, Chen et al.^53^ employed mathematical homogenisation theory to derive the effective macroscale equations. The resulting model captured the orthotropic nature of the composite material, and can be exploited to determine how local mechanical environment experienced by cells embedded within the construct^53^ depends on the composite material properties (e.g. fibre dimension and properties). In a complementary approach, Castilho et al.^54^ employed a finite element (FE) model to explore the reinforcement mechanisms of fibre-hydrogel constructs.

While the studies of Chen et al.^53^ and Castilho et al.^54^, focused on the material properties of the scaffold, techniques of mathematical homogenisation can also be employed to derive systems of homogenised partial differential equations describing tissue growth within biomaterial scaffolds^11,55,56^. Alternative routes to describing an evolving biological tissue, in which the volume fraction of the constituents/phases – cells, ECM, interstitial fluid etc - change over time utilise multiphase mixture theory, based on the principles of mass and momentum conservation with specified constitutive laws describing the interactions between the phases^57^. Such a multiphase framework has been employed in a multiscale setting to describe the properties of a tissue growing on a rigid porous scaffold: again, mathematical homogenisation techniques can used to derive effective macroscale equations that describe the effective properties of the construct, and retain explicit dependence on both the microscale scaffold structure and the multiphase tissue dynamics^58^. When considering cell-seeded construct growth within bioreactors, these bioactive multiphase models must be coupled to surrounding single phase fluid through specification of the appropriate boundary conditions^59^.

Mathematical models and computational approaches describing bioreactor processes enable identification of optimal process conditions leading to robust and economically viable products^60^. Taking a mechanistic model for the growth of neotissue in a perfusion bioreactor, Mehrian et al.^61^ applied model reduction techniques to extract a set of ordinary differential equations from the original set of partial differential equations. The simpler reduced system enabled rapid simulation, allowing the application of rigorous optimisation techniques. Bayesian optimisation was applied to find the medium refreshment regime in terms of frequency and percentage of medium replaced that would maximise neotissue growth kinetics during 21 days of culture. The simulation results indicated that maximum neotissue growth will occur for a high frequency and medium replacement percentage, supporting existing reports in the literature^61^.

### Clinical translation

Mathematical models can also be used to ask “what if…?” questions (hypothesis testing), allowing us, for example, to generate experimentally testable predictions for the way cells or engineered tissues behave after implantation. A recent theoretical study using continuum models^62^ of homoeostatic hematopoeisis put forward a novel interaction between hematopoeitic stem cells (HSCs) and niche cells, namely that niche cells could be quiescence-inducing, while the HSC in turn promote the survival of the niche cells. This mechanism would have the advantage that a large excess of niche cells can compensate large fluctuations in HSC number, unlike proliferation-inducing niche interactions. The differential equation model based on this premise was able to explain why there is a delay in HSC recovery after near-complete ablation, but not after irradiation (which kills a smaller fraction of cells). Such insights stemming from the basic regenerative biology can be exploited to make sense of the dynamics of recovery after cell transplantation, and how the ratio of niche to stem cells affects the performance of cell therapy or tissue engineering approaches.

Mathematical models can also be used for clinical optimisation of a regenerative therapy e.g. to optimise RM treatment strategies by understanding the trade-offs involved. One such trade-off is between quick repair and risk of fibrosis in ischaemia, which has been investigated using a combination of mouse experiments in a kidney injury model and a differential equation model of cell-cell communications^63^. In the model, Wnt overexpression would decrease the risk of death but increase fibrosis, while Wnt down-regulation would decrease fibrosis but increase risk of death. This led to an optimal treatment prediction of sequentially applying Wnt agonist and antagonist which ultimately could lead to increased survival and decrease fibrosis risk.

Mathematical models can assist at the end of the translational pathway, for example we can use models to gain a deeper understanding of the efficacy of treatments. In liver regeneration, mesenchymal stem/stromal cells are directed to sites of injury by SDF-1, which has potential for cell-based therapies. A differential equation model has recapitulated the in vivo response to treatment of liver injury for different SDF-1 concentrations and doses of transplanted cells^64^, including the beneficial effect of hypoxia-preconditioning to increase the CXCR4 receptor concentration.

Mathematical modelling can also give confidence to enable new protocols for RM to reach the clinic. Using clinical retrospective data, modelling can predict the importance of contributions of aspects of the protocol to the eventual outcome of the treatment. One example is theoretical modelling work for autologous Chondrocyte implantation (ACI) which is an effective treatment for cartilage defects^65,66^. From clinical and animal studies it was unclear whether the type or number of implanted cells is important. To determine the effect of the number and type of implanted cells on cartilage repair, Campbell et al.^65,66^ formulated a reaction-diffusion model for repair after implanting chondrocytes or mesenchymal stem cells (MSCs). The model captured cell migration, proliferation and differentiation, nutrient diffusion and depletion, and cartilage matrix synthesis and degradation at the defect site, both spatially and temporally. They identified that the number of implanted cells had only a marginal effect on the defect fill time or the maturation time, and that the implantation of MSCs vs chondrocytes did not affect maturation time but did affect the nature of the maturation. Chondrocyte implantation gave the most mature cartilage towards the bottom of the defect, but MSC implantation gave the most mature cartilage towards the surface of the defect. The small effect of cell number in this study may explain why both clinical and animal studies have been inconclusive in defining dosing of cells. This result gave the clinical team’s MHRA-licensed cell manufacturing facility confidence to implement wide cell release criteria with respect to cell numbers. The small maturation difference between chondrocytes and bone marrow derived MSCs agrees with experimental studies^65,66^. Chen et al.^67^ also considered a reaction-diffusion model for identifying optimal strategies for chondrogenesis in tissue engineering applications. Experimentally, a hydrogel is seeded with a layer of MSCs lying below a layer of chondrocytes, and the MSCs are stimulated from above with exogenous TGF-beta, and then cultured in vitro. Through mathematical modelling, Chen et al.^67^ identified how the initial concentration of TGF-beta, the initial densities of the MSCs and chondrocytes, and the relative depths of the two layers influence the long time composition of the tissue construct^61,67^.

These examples above demonstrate how mathematics has been used to model regenerative biology at multiple stages of the translational pathway but there are clearly further areas where the input of theoretical and computational approaches would benefit the speed of progress towards the clinic. Table 1 identifies stages in the process where help is needed to define the appropriate clinical regenerative protocol more rapidly and reproducibly. This table is intended to establish the potential for the mathematical community to contribute at each step of the translational process. Each part of the table underpins some basic biological and/or engineering question where mathematical modelling could potentially add value.

For many biomedical and clinical researchers, the concept of how to approach the relationship with mathematicians can be daunting. This review is a first step to try and provide a reference which illustrates previous work and signposts where to go for future studies. To help researchers to identify possible areas for collaboration, Table 2 identifies areas which can be modelled and specific approaches which may be or have been used in the literature. What is needed are interactive workshops, training pathways and defining some common languages to support this interaction.

## Conclusion

In this review, we have highlighted the enormous potential for embedding mathematical and computational approaches within the regenerative medicine pipeline. To successfully achieve this however requires a number of challenges to be overcome. For example, theoretical model development often lags behind experimental approaches in the earlier stages of the research, prohibiting their early use as predictive tools to guide and inform experimental design. Another challenge arises when model parameterisation is hindered by a lack of experimental data (or the right kind of experimental data). Addressing issues of structural and practical identifiability of mathematical models is key, and, in simple terms, means to check to what extent (groupings of) parameters can be determined by statistical fitting of observable data in principle or in practice. Issues of non-identifiability can then drive further model reduction and/or additional experiments.

To overcome these potential bottlenecks it is essential to have mechanisms in place to allow integrated mathematical and experimental research programmes to be designed and implemented, including interactive workshops, combined and reciprocal training pathways for wet and dry scientists, and funding schemes to engage interdisciplinary teams of mathematicians, regenerative medicine scientists, and clinicians (see also^68^).

In conclusion, the opportunity to engage mathematics within a growing regenerative medicine community has the potential to enable more rapid translation of cell-based approaches to the clinic. In contrast to laboratory experiments which are often time consuming and expensive, mathematical modelling approaches are much faster and cheaper. Embedding mathematics within regenerative medicine enables researchers to go beyond the usual trial-and-error approach, be guided in their experimental design, and therefore accelerate advances in regenerative medicine. In silico approaches can provide added value in understanding complex regeneration events in tissues in vivo and in growth environments in vitro. This review highlights the wealth of opportunities for collaboration between mathematicians and regenerative medicine scientists, and to identify where modelling approaches can contribute to the many stages of the regenerative medicine pipeline to address key challenges in translation.
